# Maximum type 1 error rate inflation in multiarmed clinical trials with adaptive interim sample size modifications

**DOI:** 10.1002/bimj.201300153

**Published:** 2014-04-22

**Authors:** Alexandra C Graf, Peter Bauer, Ekkehard Glimm, Franz Koenig

**Affiliations:** 1 Center for Medical Statistics, Informatics and Intelligent Systems, Medical University of ViennaSpitalgasse 23, 1090, Vienna, Austria; 2 Competence Center for Clinical Trials, University of BremenLinzer Strasse 4, 28359, Bremen, Germany; 3 Novartis Pharma AG, Novartis Campus4056, Basel, Switzerland

**Keywords:** Conditional error function, Interim analysis, Maximum type 1 error, Sample size reassessment, Treatment selection

## Abstract

Sample size modifications in the interim analyses of an adaptive design can inflate the type 1 error rate, if test statistics and critical boundaries are used in the final analysis as if no modification had been made. While this is already true for designs with an overall change of the sample size in a balanced treatment-control comparison, the inflation can be much larger if in addition a modification of allocation ratios is allowed as well. In this paper, we investigate adaptive designs with several treatment arms compared to a single common control group. Regarding modifications, we consider treatment arm selection as well as modifications of overall sample size and allocation ratios. The inflation is quantified for two approaches: a naive procedure that ignores not only all modifications, but also the multiplicity issue arising from the many-to-one comparison, and a Dunnett procedure that ignores modifications, but adjusts for the initially started multiple treatments. The maximum inflation of the type 1 error rate for such types of design can be calculated by searching for the “worst case” scenarios, that are sample size adaptation rules in the interim analysis that lead to the largest conditional type 1 error rate in any point of the sample space. To show the most extreme inflation, we initially assume unconstrained second stage sample size modifications leading to a large inflation of the type 1 error rate. Furthermore, we investigate the inflation when putting constraints on the second stage sample sizes. It turns out that, for example fixing the sample size of the control group, leads to designs controlling the type 1 error rate.

## 1 Introduction

In the last decade, adaptivity in clinical trials with design modifications such as sample size reassessment or treatment selection at an interim analysis has gained increasing attention. One may argue that there have always been modifications when performing clinical trials, for example simply covered by amendments to the study protocols. However, it has been shown that if, after design modifications, the critical boundaries and test statistics for the corresponding fixed sample size design are used, then the type 1 error rate is inflated. For the comparison of the means of a normally distributed outcome with known variance between a single treatment and a control in parallel groups and balanced sample sizes, that is equal sample size in the treatment and control group, Proschan and Hunsberger (1995) derived the maximum possible type 1 error rate inflation. They assumed that the experimenter, for any interim outcome, would choose the second stage sample sizes in such a way that the conditional type 1 error rate is maximized (“worst case scenario”). This strategy will also maximize the overall type 1 error rate. They showed that the type 1 error rate can be inflated from 0.05 to 0.11. Graf and Bauer (2011) extended these worst case arguments to the case of unbalanced sample size reassessment showing that the maximum type 1 error rate increases to 0.19 when the allocation ratio is allowed to change at interim. However, in this unbalanced case the maximum of the conditional type 1 error rate can only occur if the experimenter knows the value of the nuisance parameter, the common mean under the null hypothesis. This may at least approximately apply for the control treatment if a large number of data from previous experiments is available.

Many methods for type 1 error control in adaptive designs are available for testing a single hypothesis (Bauer, 1989; Bauer and Koehne, 1994; Proschan and Hunsberger, 1995; Lehmacher and Wassmer, 1999; Mueller and Schaefer, 2001; Brannath et al., 2002; Mueller and Schaefer, 2004; Gao et al., 2013) and have been applied in clinical trials. Multiarmed selection designs have been proposed (e.g. Thall et al., 1988, 1988) and have been extended to allowing for adaptive design modifications (Bauer and Kieser, 1999; Koenig et al., 2008; Bretz et al., 2009; Bebu et al., 2013; Sugitani et al., 2013). With the rise of adaptive methods in clinical trials, the main emphasis has been on strict control of the type 1 error rate to maintain the strictly confirmatory nature (EMA, 2007; FDA, 2010; Wang et al., 2013).

However, there are complaints that the adaptive machinery has become too complicated with “tests that resort to nonstandard adjustments and weightings appear mysterious to all but the specialist in adaptive design” (Metha and Pocock, 2012). From an operational perspective, adaptations put a burden on data analysts who have to clean data for interim decision making and on drug supply managers who have to deal with the possibility that doses may be added to or removed from the trial. Uncertainty at the planning stage about the total funds needed for the trial can also be a concern. From a statistical perspective, it has been argued by some experts that adaptive designs offer little advantage over more conventional group-sequential designs (Tsiatis and Metha, 2003; Jennison and Turnbull, 2006; Levin et al., 2013) and that they use test statistics that might violate desirable principles like sufficiency (Burman and Sonesson, 2006). However, these criticisms of adaptive designs are not uncontroversial themselves (Brannath et al., 2006). In any case, such additional burden may prevent experimenters from using adaptive design methodology and resort to either ignoring the issue or using seemingly simple adjustments like Bonferroni or Dunnett corrections. It is therefore desirable to investigate the maximum type 1 error inflation arising from such strategies. Regarding specific clinical trials, the precise quantification of the inflation can also be a guide to decide whether the implementation of the adaptive test machinery is really necessary, or whether a simpler adjustment might suffice, possibly after additional restrictions of the interim decision options, like upper and lower limits on the allowed sample size modifications.

In this work, we investigate the maximum type 1 error rate when *k* test treatments are compared to a single common control and when treatment selection is allowed at interim either with or without flexible sample size reassessment. Designs of multiarmed clinical trials with interim treatment selection have attracted a lot of research in the last decade (Zeymer et al., 2001; Gaydos et al., 2009; Barnes et al., 2010). Nevertheless, the number of conducted or started trials seems to be rather limited (Elsaesser et al., 2014; Morgen et al., 2014).

In Section 2011, we give a motivating example of a clinical trial where the experimenters decided to use the conservative Bonferroni procedure instead of an adaptive approach. In Section 2004, we introduce the hypothesis tests and the type of interim adaptations investigated to calculate the maximum type 1 error rate. In Section 2001, we consider the situation when the treatment with the largest observed interim effect is always selected for the second stage. Furthermore, we investigate the maximum type 1 error rate when second stage sample sizes are restricted to range within a prefixed interval. In Section 5, we mainly focus on the case of 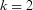 treatment arms, always proceeding with both treatments and the control to the second stage. In Section 6, we discuss our findings in the context of the motivating example and give some practical considerations. This is followed by concluding remarks in Section 7.

## 2 Motivating example

Barnes et al. (2010) give a recent case study for a two-stage clinical trial on the drug indacaterol to treat chronic obstructive pulmonary disease (COPD). This study comprised a first stage for dose-finding with dose selection after 14 days of treatment, and a second stage evaluating efficacy and safety during 26 weeks of treatment. The dose-finding stage included seven randomized treatment arms, four doses of the study drug, placebo and two further treatment groups with active comparators. At an interim analysis after the first stage the indacaterol doses were selected using preset efficacy and safety data (Lawrence et al., 2014). A multiplicity correction using a Bonferroni adjustment with 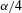 was applied, despite the fact that in the final efficacy analysis only the two selected indacaterol doses should have been compared individually against placebo based on the pooled data of both stages with prefixed sample sizes. This approach controls the type 1 error rate if the sample size, as in the given example, is prefixed. However, due to the overcorrection, this approach is conservative. The authors themselves acknowledge that the approach “is statistically somewhat conservative, but it has the merit of simplicity”. The question arises whether for such a design sample size reassessment strategies would have been possible without inflating the type 1 error rate.

## 3 Trial design

In the following, we assume that a clinical trial is designed for *k* treatment and one control arm where a two-stage design should be applied. In a first stage the observed outcome measures 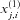 from patients 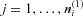, randomized to one of 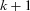 groups, that is to the control, denoted by index 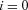, or to one of the treatment groups, 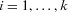 are investigated. The outcome is assumed to be normally distributed with common known variance, 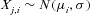. Without loss of generality we set 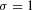. Having obtained at the end of the first stage 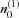 observations in the control and 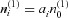, 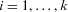 observations in the treatment groups, the sample means 
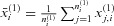
 for 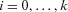 are calculated. The 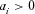 denote the prefixed first-stage-allocation-ratios between treatment group *i* and control. The experimenter may set the second stage sample sizes to 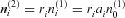 in the treatment groups and to 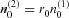 in the control group with the second-to-first-stage-ratios 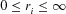, 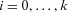 based on the interim sample means.

In the final analysis, after the second stage, we test the hypotheses 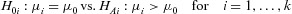
using the standardized mean difference 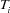 pooling the data of both stages and comparing it to the critical boundary 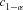 as used for the fixed sample size design. This means that adaptivity is not accounted for, neither in the test statistics nor in the critical boundary. The test statistics is defined as: 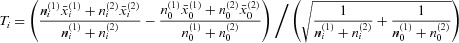
for 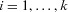 with 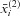, 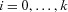 denoting the second stage sample means.

We obtain the worst case scenarios for each possible interim outcome by searching for the second-to-first-stage ratios maximizing the conditional type 1 error rate, 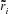. Generalizing the formula in Koenig et al. (2008) the conditional type 1 error rate for rejecting at least one treatment-control comparison in the final analysis, given the observed interim data is (see Appendix A1): 
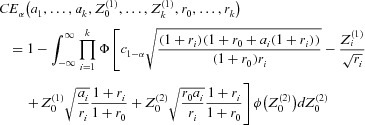
1where α is the preplanned level for the type 1 error rate and 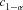 the critical boundary of the preplanned final tests (see Remark 3.1). The 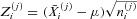, 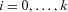, 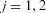 are defined as the standardized differences between the sample mean and the common true mean μ under the global null hypothesis of stage 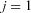 (at interim) and 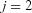, respectively (without loss of generality 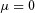). The cumulative distribution function and density of the standard normal distribution are denoted by Φ and ϕ, respectively. Note that 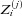 follow independent standard normal distributions.

When second stage sample sizes are not constrained, the maximum type 1 error rate is given by 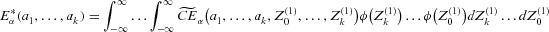
2where 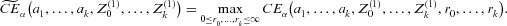


Whereas 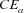 is a function of 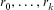, 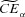 is a function of 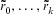, the second-to-first-stage-ratios leading to the maximum 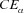. The 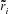 are determined for a given interim outcome 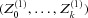 and are therefore a function of the 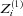. Thus, 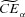 does not depend on 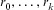.

In the following we use a quasi Newton method provided by the R-function optim for numerical optimization and for numerical integration we used the R-function integrate (R Development Core Team, 2012). R-programs to calculate the maximum type 1 error rate are available as Supplementary Information.

**Remark 3.1**

The critical boundary 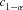 of the preplanned test may be defined in different ways: (i) as the 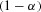-quantile of the standard normal distribution, 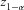, if no correction at all for multiplicity is applied or (ii) as a Dunnett critical boundary (Dunnett, 1955) based on the preplanned first-stage-allocation-ratios 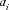, 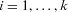 to adjust for multiplicity due to the treatment-control comparisons. Even strategy (ii) may not guarantee type 1 error control if additional sample size reassessment is performed at interim. Moreover, in case of sample size reassessment (and/or treatment selection) the Dunnett critical boundary would not be fixed a priori when calculated for the actual sample sizes in the final analysis. For simplicity, we will apply the pre-fixed Dunnett boundary, 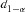, based on the preplanned first-stage-allocation-ratios 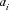 between treatment and control in the following. Remarks 4.1 and 4.2 discuss how results change if instead critical boundaries are based on actual (reassessed) total sample sizes in the final analysis.

**Remark 3.2**

For 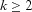 we calculate the maximum type 1 error rate under the global null hypothesis 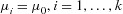. A proof that the maximum type 1 error is attained under the global null hypothesis is given in Appendix A2.

**Remark 3.3**

For 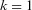, Graf and Bauer (2011) showed, by numerical evaluation, that the maximum type 1 error in the case of balanced first stage sample size between treatments before the interim analysis (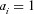, 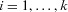) is an upper bound. For 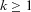 we will likewise set 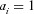, since it is the most common scenario applied in practice. Note that for many-to-one comparisons, the scenario with 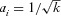 leads to the smallest required sample size for a given power and significance level. Therefore we will also give some numerical results for this allocation ratio.

## 4 Selection of the most promising treatment at interim

We first consider that in the interim analysis the treatment group *m* with the largest observed interim effect 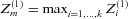 is selected for the second stage, setting 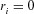 for 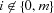. The second-to-first-stage-ratios 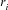, 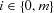 may be set based on the interim results, 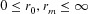. In the final analysis, only the selected treatment group *m* is compared to the control group (using data of both stages). The corresponding null hypothesis 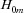 is rejected, if the final test statistic 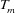 exceeds the critical value 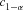. Note that the maximum type 1 error rate for the case of always selecting the best treatment is an upper bound for the maximum type 1 error rate when in a particular trial another single treatment is selected, for example the treatment with the second largest observed effect at interim because of potential safety issues for the most effective treatment. Clearly, under the global null hypothesis and for balanced first stage sample sizes over the *k* treatments, selecting a treatment with an interim effect smaller than the largest observed interim effect will reduce the maximum type 1 error rate. Following the lines of Graf and Bauer (2011), the conditional type 1 error rate 1994 for this scenario simplifies to 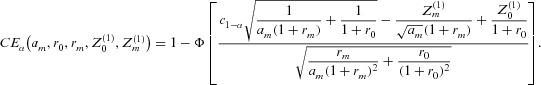
3Note that if the treatment with the largest observed interim effect is selected, *m* is random and therefore also 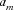 is a random variable. In the following we set 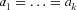 so that 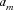 is no longer a random variable and the maximum type 1 error rate can be evaluated by 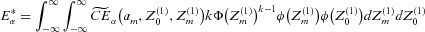
4where 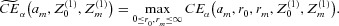
5and 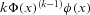 is the probability density function of the maximum of independent standard normal distributions.

### 4.1 Equal second-to-first-stage-ratios

Let 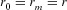 with 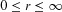, that means only allowing for equal second-to-first-stage ratios, and let furthermore 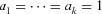 indicating balanced first stage sample sizes for the treatment and the control groups. After the second stage, the selected treatment group is compared to the control group (using data of both stages) applying the critical value 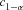 of the pre-planned test. Note that for this scenario the final test is balanced between both groups. In a slight modification of Proschan and Hunsberger (1995), the conditional type 1 error rate 2013 of the final treatment-control comparison for 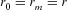 and 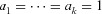 reduces to 
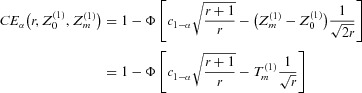


For notational convenience, the first stage test statistics 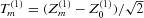 is used. The conditional type 1 error rate does not depend on the unknown nuisance parameter μ.

Calculation of 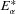 in this balanced case follows the lines of Proschan and Hunsberger (1995). The essential difference is that the density of the maximum of *k* independent standard normal distributions has to be used in the integration. The subspaces of the interim sample space to perform separate optimizations remain the same (see Appendix A3).

**Figure 1:**
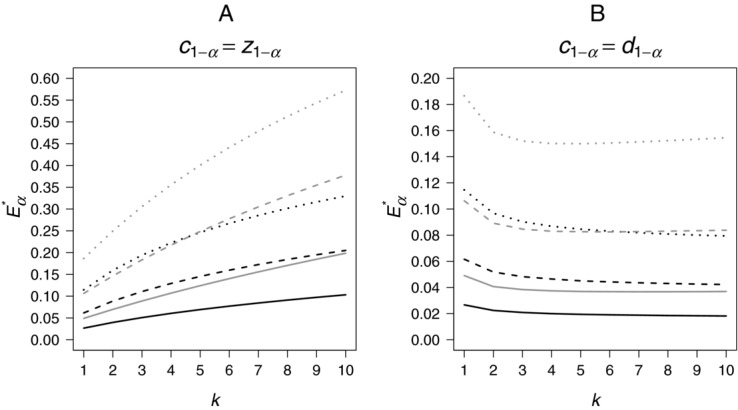
Maximum type 1 error rate 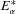 when always selecting the treatment with the maximum effect at interim for an increasing number of treatment groups *k*. Results are given when using the uncorrected critical boundary 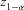 (A) or the fixed Dunnett critical boundary 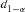 (B) for equal (black lines) and flexible (gray lines) second-to-first-stage ratios. Nominal one-sided α was set to 0.01 (solid lines), 0.025 (dashed lines), and 0.05 (dotted lines).

The black lines in Fig.1 show that if no correction for multiplicity is done (Fig.1A), the type 1 error is highly inflated and increases with *k*. Using Dunnett boundaries for *k* treatment-control comparisons, that means adjusting for all initially planned comparisons (Fig.1B), the overall type 1 error decreases with *k*, that means correcting for multiplicity of all possible individual treatment-control comparisons leads to a smaller inflation of the overall type 1 error as compared to 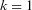. For increasing *k*, the correction is done for an increasing number of 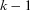 hypotheses not tested in the final analysis. Correcting for all possible individual treatment-control comparisons would be a conservative approach if the second stage sample size would be fixed independently of the data, for example in the planning phase. Here the inflation of the maximum type 1 error rate is caused by the worst case sample size reassessment rule.

For a direct comparison with the case of no treatment selection discussed later (see Section 5), the columns “equal” in Table1 show the maximum overall type 1 error rate for 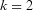 with and without correction for multiplicity as well as for the case of 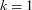 (Proschan and Hunsberger, 1995).

**Table 1:** Maximum type 1 error rate for 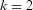 with and without treatment selection, with or without adjustment for multiplicity and with equal or flexible second-to-first stage ratios as compared to the case 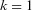

nominal α	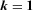			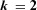		
	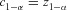		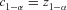		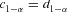	
treatment selection of most promising treatment						
	equal (Proschan and Hunsberger, 1995)	flexible (Graf and Bauer, 2011)	equal (Section 4.1)	flexible (Section 4.2)	equal (Section 4.1)	flexible (Section 4.2)
0.01	0.0267	0.0491	0.0398	0.0697	0.0224	0.0407
0.025	0.0616	0.1064	0.0887	0.1466	0.0518	0.0892
0.05	0.1146	0.1867	0.1594	0.2496	0.0968	0.1588
without treatment selection						
	equal (Proschan and Hunsberger, 1995)	flexible (Graf and Bauer, 2011)	equal (Section 5.1)	flexible (Section 5.2)	equal (Section 5.1)	flexible (Section 5.2)
0.01	0.0267	0.0491	0.0478	0.0800	0.0263	0.0473
0.025	0.0616	0.1064	0.1058	0.1701	0.0610	0.1037
0.05	0.1146	0.1867	0.1897	0.2885	0.1138	0.1842

If the first-stage-allocation-ratios are set to 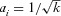, 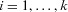, a smaller maximum type 1 error rate was found. When using the Dunnett critical boundary, for 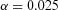 the values are 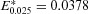 for 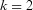 and 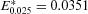 and 0.0340 for 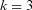 and 4, respectively. For comparison, setting 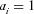, the values are 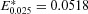 (see Table1 and Fig.1), 0.0482 and 0.0463 (see Fig.1) for 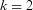, 3, and 4, respectively. Similar results can be found for 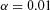 and 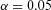.

**Remark 4.1**

To give an impression of how results may change if the actual final adapted sample sizes are used in the calculation of the critical Dunnett boundaries (see Remark 3.1) for 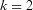, the values would become only slightly smaller than in Table1: 0.0221, 0.0507, and 0.0948 for 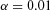, 0.025, and 0.05, respectively.

### 4.2 Flexible second-to-first-stage-ratios

“Flexible” second-to-first-stage ratios allow different sample size reassessments for the selected treatment and the control, for example a sample size decrease for the control, but a sample size increase for the selected treatment group. For each interim outcome, the worst case 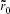 and 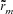 may differ. The sample size of the final treatment-control comparison may then be unbalanced between treatment arms. If we again assume balanced first stage sample sizes, the conditional type 1 error rate is now calculated by (3) setting 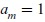. We use the independence of 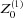 and 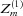 to get rid of the nuisance parameter μ. The conditional type 1 error rate cannot be written as a function of the test statistic 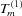 as in Section 4.1. As in Graf and Bauer (2011), the calculation of 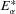 can be separated into several parts of the interim subspace using 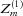 instead of 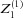. To evaluate the maximum type 1 error rate we partition the interim subspace in a way analogous to Graf and Bauer (2011) (see Section 2009 in the Supplemental Materials).

The gray lines in Figs.1A and B show that allowing for flexible second-to-first-stage ratios substantially increases the possible maximum type 1 error rate. Using 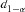 (Fig.1B) in all scenarios leads to a nonmonotonous behavior with respect to the number of treatments *k*. An explanation for this is that the fixed boundaries are correct for the worst case scenarios, where the overall sample size is balanced between treatment and control, whereas for the unbalanced worst case scenarios they lead to smaller critical boundaries as compared to the boundaries using the actual total sample sizes. For larger *k* this difference in the correlation matrices is extended to all the 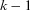 dropped treatments at interim, so that the differences between unbalanced and balanced critical boundaries tend to increase with increasing *k* which in the end leads to an increase in the maximum type 1 error rate. Again, to allow a direct comparison to the other discussed scenarios, the columns “flexible” in Table1 show the values for 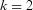 for both choices of the critical boundary as well as 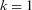 (Graf and Bauer, 2011).

If 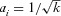, 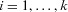, as in the case of equal second-to-first stage ratios, a smaller maximum type 1 error was found. When using the Dunnett critical boundary, for 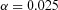 the values are 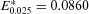, 0.0792, and 0.0753 for 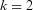, 3, and 4, respectively. For comparison, setting 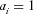, the values are 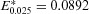 (see Table1 and Fig.1), 0.0846, and 0.0830 (see Fig.1) for 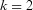, 3, and 4, respectively. Similar results can be found for 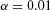 or 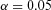.

**Remark 4.2**

When using Dunnett critical boundaries as in Remark 4.1, the maximum type 1 error rate up to 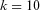 (data not shown) is smaller than for Dunnett critical boundaries based on balanced sample sizes. The maximum type 1 error rate is decreasing in *k* and hence also differences between the two approaches increase with *k*.

### 4.3 Constrained second stage sample size

**Figure 2:**
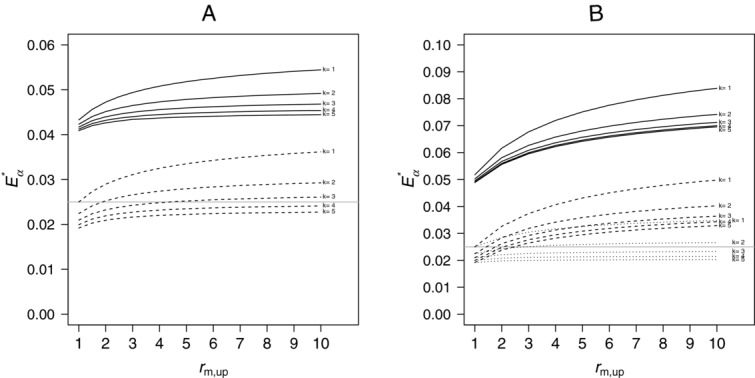
Maximum type 1 error rate 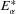 as a function of the upper boundary 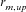 for the second-to-first-stage-ratio when always selecting the treatment with the maximum effect at interim for constrained second stage sample size for equal (A) and flexible (B) second-to-first-stage-ratios using Dunnett corrected critical boundaries. Solid lines: 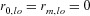, 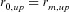, dashed lines: 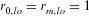, 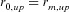 and dotted lines: 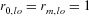, 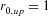. Nominal one-sided α was set to 0.025.

Unconstrained sample size reassessment of course will hardly be used in practice. We therefore put constraints on the second-to-first-stage-ratios 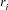, 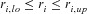, 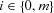. The ranges for the maximization in formula 2002 are therefore changed to 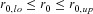 and 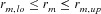. Figure2 shows the maximum type 1 error rate 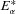, 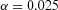 for different constraints on sample size reassessment using the Dunnett critical boundary 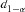:

1. I 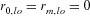, 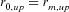, 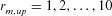: Setting the lower boundary to 0 means that we allow for early rejection at interim. The solid lines in Fig.2 show that 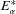 is increasing with increasing upper boundary, flattening off for larger values. Allowing for flexible second-to-first-stage-ratios (solid lines in Fig.2B), the increase with the upper boundary is even steeper than for equal ratios (Fig.2A). However, the results for 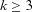 are very similar.
2. II 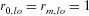, 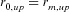, 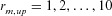: In this scenario, the second stage sample size has to be at least as large as the first stage sample size for the selected treatment and the control. The dashed lines in Fig.2A. show that for equal ratios and 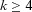, the maximum type 1 error is always below the nominal 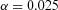. Calculations including numerical integration of 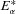 for 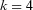 and 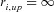 give a value of 0.02509. Therefore, for 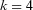 selecting always only one treatment and the control, such type of constraints may be safely applied in practice without inflating the type 1 error rate. The reason is that there is a tradeoff (i) between the overcorrection from using Dunnett boundaries adjusting for treatment-control comparisons that are not carried over to the final test and (ii) the inflation due to data-dependent choice of the final sample size of the selected treatment (equal ratios, total sample size per selected treatment at least twice the first stage sample size per group). The smaller the prefixed range for the second stage sample sizes the smaller the impact of the latter effect.
  Similar results can be found for a nominal α of 0.05 and 0.01. For 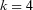 and 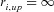 the values are 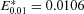 and 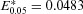. Note that in the scenario for 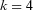 without any interim sample size reassessment, for example: 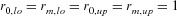, the selection of one treatment and the control would happen quite late in the trial in terms of total sample over all groups (at a fraction of 5/7).
  Allowing for flexible second-to-first-stage-ratios (Fig.2B) only for smaller windows (smaller 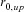 and 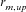) 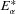 does not exceed α. For example for 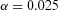 and 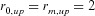, the number of treatments has to be larger than 3 so that 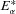 will always be below 0.025.
3. III 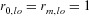, 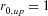, 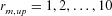: In this case, the second-to-first-stage-ratios are allowed to be flexible by definition, the only option for sample size adaptation is the choice of a second stage sample size for the selected treatment to be at least as large as in the first stage and not to exceed 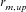 (see dotted lines in Fig.2B). Such an adaptation may arise from a rare adverse event in the selected treatment group requiring additional information. It is interesting to note that for 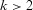 the maximum type 1 error rate 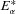 will never exceed the nominal level, even if the upper boundary is set to ∞. For 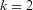 no inflation occurs with 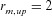. Similar results can be found for a nominal α of 0.05 and 0.01. Note that Fig.2B shows that the type 1 error rate is not inflated when Dunnett critical boundaries are used in case of an allocation ratio to control of 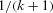 between treatment(s) and control in both stages, that is 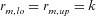.

## 5 No treatment selection at interim

Since selecting only the treatment with the largest interim effect is a natural strategy often discussed in the literature (Cohen and Sackrowitz, 1989; Bowden and Glimm, 2008; Friede and Stallard, 2008; Stallard et al., 2008; Bauer et al., 2010), we first elaborated on this in Section 2001. However, if all initially planned treatment arms are further investigated in the second stage, under the global null hypothesis, the maximum type 1 error rate is larger than for any other case with treatment selection. The reason is that dropping treatments at the interim analysis can be viewed as a constrained sample size reestimation problem (with 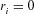 or 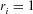 as the only options for treatment *i*), and this cannot produce a larger maximum of the conditional type 1 error rate than the unconstrained optimization problem.

For 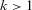 we were not able to find a general closed solution for the maximum type 1 error rate (even if a single constant 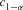 is used as a critical boundary for all the *k* standardized treatment vs. control test statistics). To put the above optimization problem into a manageable framework, we illustrate the calculation for the case of two experimental treatment arms (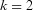) in the following. For the less complex scenario of equal second-to-first-stage ratios, numerical results are reported for 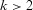.

### 5.1 Equal second-to-first-stage-ratios

As an extension to Proschan and Hunsberger (1995) we first investigate the case of equal second-to-first-stage-ratios setting 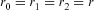. Assuming furthermore that the first stage sample sizes are balanced, that is setting 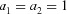 (and therefore also that the final stage sample sizes are balanced between treatment arms), for 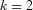 formula 1994 simplifies to 
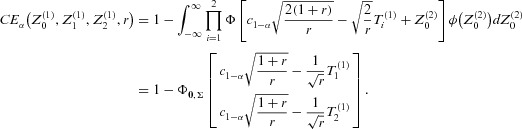
As in Section 4.1, for notational convenience, the first stage test statistics 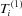 for comparing treatment *i* to the control are used. The conditional type 1 error rate does not depend on the nuisance parameter μ. The cumulative distribution function of the multivariate normal distribution with two-dimensional mean zero-vector 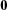 and covariance-matrix Σ with elements 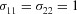 and covariance 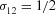 is denoted by 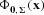. To calculate the worst case conditional type 1 error rate we have to partition the 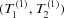-plane.

1. I If 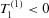 and 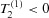 the largest conditional type 1 error rate is obtained by setting 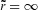, 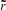 denoting the worst case second-to-first-stage ratio. The second stage is now overruling the negative interim effect and therefore yielding a 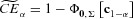 that is equal to α if 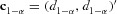. Since 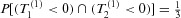 for the bivariate normal distribution with 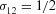 (see e.g. Kotz et al., 2000), the contribution of this subspace to the overall maximum type 1 error rate 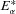 is 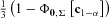.
2. II If 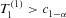 or 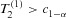 the largest conditional type 1 error rate 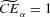 (applying early rejection at interim and setting 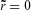) is obtained. This leads to a contribution to 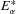 of 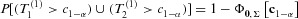 that is equal to α if 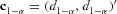.

In the remaining interim subspace we were not able to find a closed solution for 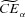. Therefore, we used numerical optimization of the single parameter *r*. The “equal”-columns of Table1 show the results for the overall 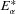 for the case of 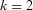, with and without correction for multiplicity. As is to be expected, applying the naive unadjusted critical boundary 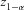 may result in a further considerable type 1 error rate inflation as compared to 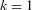. An interesting finding is that when using the Dunnett critical value, 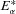 are close to the results for 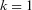.

For 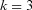 and using Dunnett critical boundaries for 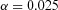 the maximum type 1 error rate is still inflated up to 0.0545, but interestingly the inflation is smaller compared to 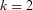. For 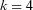 treatments, 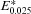 is flattening off at an inflated level of 0.0543. For 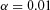 and 0.05 the same tendencies can be found.

### 5.2 Flexible second-to-first-stage-ratios

If we allow for flexible second-to-first-stage-ratios, we again have to use the independent 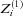 (instead of the test statistics 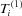) to get rid of the nuisance parameter μ. If we assume balanced first stage sample size, the conditional type 1 error rate is now calculated by 1994 setting 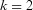 and 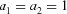. To explain the worst case scenarios in more detail, we will focus on the subspaces in terms of the interim outcome of the control group 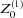.

A Subspace (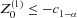): 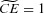 is obtained by setting either 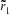 or 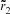 to ∞ and 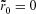. The contribution of this subspace to 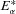 therefore is 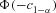.
B Subspace (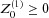): The worst case choice is setting 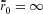 in the final analysis, getting two independent tests against the asymptotically fixed mean 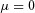. Hence the conditional type 1 error rate reduces to 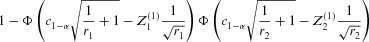
independent of 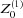. A detailed explanation for the calculation of the maximum type 1 error rate for this subspace B is given in Section 2011 in the Supplemental Materials. Summing up the results for 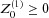, the contribution to the overall maximum type 1 error rate can be calculated by 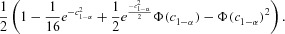
C Subspace (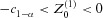): In this region the worst case conditional type 1 error rate depends on all three interim values of control and treatment groups, respectively. If either 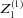 or 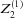 is larger than 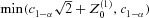 again a conditional type 1 error rate of 1 can be achieved. For the remaining regions we used numerical point-wise optimization and integration for calculating the contribution to the overall type 1 error rate 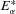.

The columns “flexible” for 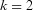 of Table1 show the total 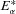 for flexible second-to-first-stage-ratios applying critical boundaries 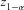 or 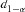. Without any correction for multiplicity (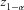), the maximum type 1 error is clearly increased as compared to the case 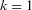. Interestingly, as for the results of equal second-to-first-stage ratios (see Section 5.15.1), when using the pre-specified Dunnett critical boundary, 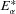 is close to the results for 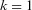.

Due to the numerical burden we did not calculate the maximum type 1 error rate for 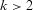. However, we expect similar findings as for the case of equal second-to-first-stage ratios at least for 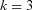 and 4, that is the maximum type 1 error rate sightly decreasing when using a Dunnett adjusted critical boundary.

## 6 Practical recommendations

The results presented for the case of selecting the most promising hypothesis at interim are of great practical interest, because they demonstrate that, given certain restrictions on the second stage sample size, naive strategies may even lead to an adequate control of the type 1 error rate. For example, if the sample size per treatment group in the second stage is at least as large as in the first stage and we only allow for equal second-to-first-stage-ratios, no inflation of the type 1 error rate occurs for the number of treatments 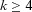 when simply using the Dunnett critical boundaries. For 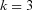, no inflation occurs when restricting the second-stage sample size to be at maximum 4 times the first-stage sample size (see Fig. A). If we fix the overall sample size in the control group, allowing for any choice of the overall sample size in the selected treatment group that increases its first stage sample size more than twofold does not lead to an inflation of α for 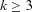 (see Fig. 2B). Therefore, if in the case study of Barnes et al. (2010) (see Section 2011) only the selection of a single treatment group and control had been pre-specified, the experimenter would have been permitted to do any balanced increase of the sample size, even when using the conventional test statistic and the less conservative Dunnett critical boundary (instead of the applied Bonferroni adjustment) for final testing. If a flexible sample size reassessment for the second stage would have been allowed for (as in Section 4.2), no type 1 error inflation would have occurred if the second stage sample size would have been constrained to be between the first-stage and twice the first stage sample size. However, it has to be noted that for realistic scenarios (as e.g. an upper bound of twice the first stage sample size) and a larger *k*, the obtained maximum type 1 error rate may be much smaller than α so that even using the Dunnett critical boundaries would lead to conservative procedures. Note that these results only apply when using prespecified-binding constraints on the selection rules.

Allowing for early rejection at interim, the maximum type 1 error rate will always be inflated. In such scenarios, if the use of conventional test statistics is preferred, one may adjust the critical boundary so that the maximum type 1 error rate is controlled. As an example, assume that we only allow for equal second-to-first-stage ratios setting the upper bound of the second-stage-sample size of the selected treatment and control to be twice the first stage sample size. For 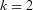 an adjusted level of 0.013 (instead of 0.025) has to be used to control the maximum type 1 error rate. In more detail, if we assume for both treatments an effect size of 0.5 times the standard deviation, a sample size of 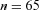 per group would be needed to achieve 80% power. Compared to a fixed sample size test with Dunnett adjusted critical boundaries, this would be a 20.4% increase of the per-group sample size. For increasing *k*, this is only slightly decreasing: for 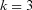 an increase of 18.8% and for 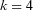 an increase of 17.0% of the per-group sample size is needed to control the maximum type 1 error rate when additionally allowing for the given sample size reassessment. To achieve a power of 90%, a slightly smaller increase in the per-group sample size is needed, that means an increase of 16.4%, 16.7%, and 15.6% would be needed for 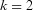, 3, and 4, respectively. All these examples show that adjusting for the worst case would be a rather conservative strategy and adaptive tests should be implemented instead (Koenig et al., 2008; Bretz et al., 2009).

## 7 Discussion

In this paper, we have investigated the maximum type 1 error rate arising from the application of a nonadaptive test used by experimenters who freely adapt their ongoing trials. This problem has been addressed by Proschan and Hunsberger (1995) for the comparison of one treatment with a control and balanced sample sizes before and after the adaptive interim analysis. They considered a restricted rule incorporating a stopping for futility criterion. This leads to procedures where the effect of adjusting the adaptation of the sample size is no longer dramatic. Graf and Bauer (2011) have extended the worst case calculations allowing for unbalanced sample sizes. In this paper, a further level of complexity has been added by considering multiple comparisons of *k* treatments with a single control. For the case without selection of a treatment arm at interim, we calculate the maximum type 1 error rate for 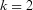 in the case of equal and flexible second-to-first-stage-ratios (assuming balanced first stage sample sizes). Not surprisingly, when applying uncorrected level α treatment-control comparisons, the worst case type 1 error is dramatically inflated. By using Dunnett-adjusted critical boundaries, the worst case inflation is still large. Interestingly, the inflation is very similar to the case of comparing 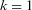 treatment to a control (Graf and Bauer, 2011). This means that when adjusting for the number of treatments for 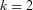, no noticeable further maximum inflation of the type 1 error rate occurs as compared to 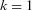.

The case of equal and flexible second-to-first-stage ratios was investigated for scenarios where only a single treatment and the control are selected at the interim analysis. In this scenario, there is a trade-off between inflation due to sample size reassessment and the overcorrection for the 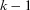 treatments finally not selected and not tested in the statistical analysis. For equal ratios, the maximum type 1 error is monotonically decreasing with *k* with a finite limit noticeably larger than the nominal level α. As expected, the impact of flexible ratios is more severe, the maximum inflation of the actual level α, though decreasing for small *k*, is increasing with larger *k*.

There are several caveats to be mentioned here. First, for the case of flexible ratios the conditional error can only be calculated when the nuisance parameter, the common mean under the global null hypothesis, is known. Secondly, the maximum type 1 error only occurs if the experimenters apply the worst case sample size reassessment rule (maximizing the conditional type 1 error rate) at any point in the interim sample space. Thirdly, in some interim subspace, the maximum is assumed if some of the second stage sample sizes go to infinity. Although theoretically interesting, this of course means that these maximum type 1 error rates can never be reached in real clinical trials. Adjusting for these “unrestricted worst cases” would be an extremely conservative strategy and cannot be recommended for use in practice. Therefore, we also investigated maximum type 1 error rates that arise when the second stage sample sizes are constrained by upper and lower limits. Some of these results are practically interesting, because they demonstrate that in certain cases, when putting restrictions on the second-stage-sample sizes, naive strategies can control the type 1 error rate. Such calculations under constraints could replace simulations of the type 1 error rate in designs with adaptive selection rules, the latter being considered problematic by some researchers (Posch et al., 2011).

Open research problems are at present the unconstrained optimization for 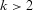, which imposes a burden of numerical integration and optimization. For the unconstrained scenario of 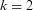, the optimization lasts up to one half second for one grid point on an Intel(R)Core(TM)i5 CPU M540 processor with 2.53GHz and it is therefore still a time consuming numerical challenge to derive a sufficiently narrow grid over the three dimensional interim subspaces with sufficiently accurate values of the maximum conditional error functions to be integrated. Also scenarios where the selection of *s*, 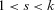 out of *k* treatment groups and the control are prespecified are of high interest.

As a conclusion, we do not recommend the use of unrestricted “worst case” adjustments since they will be far too conservative for serious consideration. If limits on sample size modifications can be imposed, it is still important to compare the operating characteristics of adaptive designs with the maximum-type-1-error-based adjustments discussed here. Only then we can decide whether sample size limits can or should be imposed and how tight they might be.

## Appendix

### A.1. Calculation of the conditional type 1 error rate

In the following we assume that the global null hypothesis applies ( for ). The conditional type 1 error for rejecting at least one treatment-control comparison in the final analysis, given the interim data, can be calculated as follows

In the final analysis after the second stage each test (comparing treatment *i* to the control group) is based on the following global test statistic:

where , , , have independent standard normal distributions. If the overall test statistic  is larger than the critical boundary  we get a false positive decision, which leads to the following inequality:

leading to

Since  and  have independent standard normal distributions for every set of values , *r*_0_, ,  and  (and hence are independent of these quantities), the conditional error can be written as in formula (1).

### A.2. The maximum type 1 error rate is attained under the global null hypothesis

For  the maximum type 1 error rate is attained under the global null hypothesis . To see this, assume (without loss of generality) that the null hypothesis is not true for *H*_01_. Then the conditional error for rejecting at least one true hypothesis is calculated in the same way as  from formula 1994, but the product going from 2 to *k* rather than 1 to *k*, further on denoted by . Obviously, for given , . This statement is true since the integrated term in formula 1994, for given , is the same for  and  but *r*_1_ does not appear in . Since the integrated function in 1994 is at every point larger (or equal) in , the whole integral must be larger (or equal) for  as compared to  for every given constellation of .

Let now denote  the second-two-first-stage-ratios leading to the maximum . Due to the above arguments

for all *r*_1_. The ratios  may not be the ratios maximizing , but finding the ratios leading to  can only increase the conditional type 1 error  and thus

This domination also holds for the integral for the type 1 error  in formula 1999, showing that the global null indeed gives the parameter constellation leading to the largest type 1 error inflation.

### A.3. Maximum conditional type 1 error rate when selecting the most promising treatment for the scenario of equal second-to-first-stage-ratios

Following the lines of Proschan and Hunsberger (1995) the maximum conditional type 1 error rate when selecting the treatment with the largest observed interim effect for the scenario of equal second-to-first-stage-ratios (refer to Section 4.1) can be calculated by dividing the interim sample space into three subspaces:

1. I If  (equivalently ) the worst case arises from setting  so that the second stage overrules the first stage adverse effect leading to  This results in a contribution to  of  since ,  here denoting the *k*-dimensional zero vector and Σ the *k*-dimensional covariance matrix with  and  for .
2. II Within the subspace  (or equivalently ), Proschan and Hunsberger (1995) showed that  is leading to a worst case conditional type 1 error rate . We found no simplification of the two-dimensional integration in this subspace.
3. III If  (or equivalently ) the test can be rejected already at interim ( with ) leading to a contribution of this subspace of  where  is a *k*-dimensional vector with values  which is  and reduces to α when using the multiplicity corrected Dunnett critical boundary .
