# Supplementary material for: Maximum type 1 error rate inflation in multiarmed clinical trials with adaptive interim sample size modifications
Source: Biom J. 2014 Apr 22;56(4):614–30. doi: 10.1002/bimj.201300153 (PMC4282114; doi:10.1002/bimj.201300153)
Supplement: Supplementary file 1 [file bimj0056-0614-SD1.pdf]

Supplemental Material to:
Maximum type 1 error rate inflation in multi-armed clinical trials with adaptive interim sample size modifications

Alexandra C. Graf,[a,b], Peter Bauer[a], Ekkehard Glimm[c] and Franz Koenig[a,1]

[a]Section for Medical Statistics, Medical University of Vienna,
Spitalgasse 23, Vienna, Austria
[b]Competence Center for Clinical Trials, University of Bremen,
Linzer Strasse 4, 28359 Bremen, Germany
[c]Novartis Pharma AG, Novartis Campus, 4056 Basel, Switzerland

1 Maximum conditional type 1 error rate when selecting the most promising treatment for the scenario of flexible second-to-first-stage-ratios

The maximum conditional type 1 error rate when selecting the treatment with the largest observed interim outcome for the scenario of flexible second-to-first-stage-ratios (refer to Section 4.2 in the main document) can be calculated by dividing the interim sample space into the following subspaces (following the lines of Graf and Bauer , 2011):

I. If $Z_0^{(1)} < -c_{1-\alpha}$ the worst case is to set $\tilde{r}_0 = 0$ and $\tilde{r}_m = \infty$ which leads to a $\widetilde{CE} = 1$. The integration can easily be applied resulting in $(1 - \Phi(c_{1-\alpha}))$. If no correction for multiplicity is done $(c_{1-\alpha} = z_{1-\alpha})$, this reduces to α.
II. In the subspace where $Z_m^{(1)} > c_{1-\alpha}$ and $Z_0^{(1)} > -c_{1-\alpha}$ similar arguments as in I. can be applied to get $\widetilde{CE} = 1$. The integration can be simplified to $\Phi(c_{1-\alpha})(1 - \Phi(c_{1-\alpha})^k)$. If no adjustment for multiplicity is done, this reduces to $\alpha(1 - \alpha^k)$.
III. If $Z_0^{(1)} > 0$ and $Z_m^{(1)} < 0$ it turned out, that setting $\tilde{r}_0 = \tilde{r}_m = \infty$ leads to $\widetilde{CE} = 1 - \Phi(c_{1-\alpha})$. The integration in this area can be simplified to $(1 - \Phi(c_{1-\alpha}))\frac{1}{2^{k+1}}$ since $P[(Z_m^{(1)} < 0) \cap (Z_0^{(1)} > 0)] = \frac{1}{2^{k+1}}$ reducing to $\alpha\frac{1}{2^{k+1}}$ if no correction for multiplicity is done.
IV. If $-c_{1-\alpha} < Z_0^{(1)} < 0$ and $-\infty < Z_m^{(1)} < 0$ it can be shown for a pre-fixed critical value that $\widetilde{CE} = 1 - \Phi\left(\sqrt{(c_{1-\alpha})^2 - (Z_0^{(1)})^2}\right)$. First, performing the integration over $Z_0^{(1)}$ along the arguments of Proschan and Hunsberger (1995) and than over $Z_m^{(1)}$ results in $\left[e^{\frac{(-c_{1-\alpha})^2}{2}} - 2\left(1 - \Phi(c_{1-\alpha})\right)\right]\frac{1}{2^{k+2}}$.

[1]Corresponding author: e-mail: franz.koenig@meduniwien.ac.at, Phone: +43-140400-7480, Fax: +43-140400-7477

V. If $Z_0^{(1)} > 0$ and $0 < Z_m^{(1)} < c_{1-\alpha}$ it can be shown that $\widetilde{CE} = 1 - \Phi\left(\sqrt{c_{1-\alpha}^2 - (Z_m^{(1)})^2}\right)$. The integration can be simplified to

$$\frac{1}{2}\int_0^{c_{1-\alpha}} \left[1 - \Phi\left(\sqrt{(c_{1-\alpha})^2 - (Z_m^{(1)})^2}\right)\right] k\Phi(Z_m^{(1)})^{k-1}\phi(Z_m^{(1)})dZ_m^{(1)}.$$

VI. It remains the area $(0 < Z_m^{(1)} < c_{1-\alpha}$ and $-c_{1-\alpha} < Z_0^{(1)} < 0)$ where

$$\int_{-c_{1-\alpha}}^0 \int_0^{c_{1-\alpha}} \widetilde{CE}(Z_m^{(1)}, Z_0^{(1)})k\Phi(Z_m^{(1)})^{k-1}\phi(Z_m^{(1)})\phi(Z_0^{(1)})dZ_m^{(1)}dZ_0^{(1)}$$

Here numerical optimization has to be used. Note however, that if $Z_m^{(1)} > \sqrt{2}c_{1-\alpha} + Z_0^{(1)}$ the worst case conditional type 1 error rate $\widetilde{CE} = 1$ can be obtained by setting $\tilde{r}_0 = \tilde{r}_m = 0$.

2 Maximum conditional type 1 error rate for the scenario of flexible second-to-first-stage-ratios for $k = 2$ and $Z_0^{(1)} \geq 0$

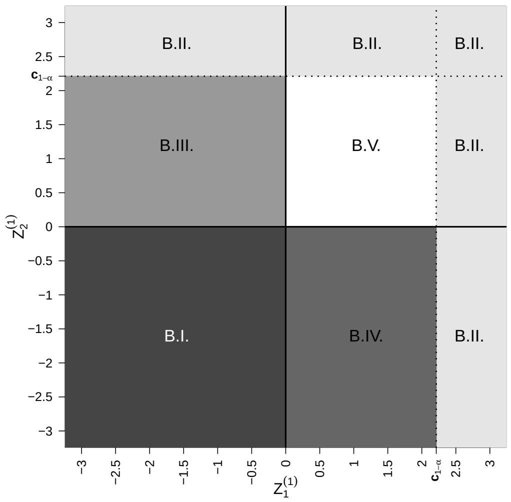

Figure 1: Subspaces of the interim outcome of treatment 1 and 2 given $Z_0^{(1)} \geq 0$ to be used for evaluating the worst case conditional type 1 error rates in case of flexible second-to-first-stage ratios.

The maximum conditional type 1 error rate for the scenario of $k = 2$ treatment-control comparisons and flexible second-to-first-stage ratios (refer to Section 5.2. B. in the main document) for the interim subspace where $Z_0^{(1)} \geq 0$ can be calculated by dividing this subspace to five parts. Figure 1 shows the partitions (B.I to B.V) in the $(Z_1^{(1)}, Z_2^{(1)})$-plane given $Z_0^{(1)} \geq 0$ where separate optimization has to be performed.

B.I. If both $Z_1^{(1)}$ and $Z_2^{(1)} < 0$ (Area B.I in Figure 1) the $\widetilde{CE}_\alpha = 1 - \Phi(c_{1-\alpha})^2$ can be yielded by setting $\tilde{r}_1 = \tilde{r}_2 = \infty$. Two-dimensional integration over this area results in a contribution to E_α^* of $(1 - \Phi(c_{1-\alpha})^2)/4$.
B.II. For $Z_1^{(1)} > c_{1-\alpha}$ or $Z_2^{(1)} > c_{1-\alpha}$ (Area B.II. in Figure 1), $\widetilde{CE}_\alpha = 1$ (final rejection) is yielded by setting $\tilde{r}_1 = 0$ or $\tilde{r}_2 = 0$. The interim effect of either treatment 1 or 2 then is tested against the asymptotically fixed $\mu = 0$ (the control group having infinite sample size). Integration over Area B.II. results in $1 - \Phi(c_{1-\alpha})^2$ which is $P[(Z_1^{(1)} > c_{1-\alpha}) \cup (Z_2^{(1)} > c_{1-\alpha})]$.
B.III. and IV. If $0 \leq Z_2^{(1)} < c_{1-\alpha}$ and $Z_1^{(1)} < 0$ (Area B.III, Figure 1) the worst case is $\tilde{r}_1 = \infty$ and $\tilde{r}_2 = \frac{c_{1-\alpha}^2 - (Z_2^{(1)})^2}{(Z_2^{(1)})^2}$, similar to Proschan and Hunsberger (1995). Hence, $\widetilde{CE}_\alpha = 1 - \Phi(c_{1-\alpha})\Phi\left(\sqrt{c_{1-\alpha}^2 - (Z_2^{(1)})^2}\right)$. By symmetry arguments, for $0 \leq Z_1^{(1)} < c_{1-\alpha}$ and $Z_2^{(1)} < 0$ (Area B.IV.) the worst case conditional error is $\widetilde{CE}_\alpha = 1 - \Phi(c_{1-\alpha})\Phi\left(\sqrt{c_{1-\alpha}^2 - (Z_1^{(1)})^2}\right)$.

Integration over this part results in

$$\Phi(c_{1-\alpha}) - \frac{1}{2}(1 + \Phi(c_{1-\alpha})^2) + \frac{1}{4}\Phi(c_{1-\alpha})e^{-\frac{c_{1-\alpha}^2}{2}}$$

using analogous arguments as in the Appendix of Proschan and Hunsberger (1995).
B.V. If both $0 \leq Z_1^{(1)}, Z_2^{(1)} < c_{1-\alpha}$ (Area V. in Figure 1) the worst case second-to-first-stage ratio $\tilde{r}_i$ can be separately derived for both treatment groups along the lines of Proschan and Hunsberger (1995) arriving at

$$\widetilde{CE}_\alpha = 1 - \Phi\left(\sqrt{c_{1-\alpha}^2 - (Z_1^{(1)})^2}\right)\Phi\left(\sqrt{c_{1-\alpha}^2 - (Z_2^{(1)})^2}\right)$$

resulting in a contribution of this subspace of

$$\left(\Phi(c_{1-\alpha}) - \frac{1}{2}\right)^2 - \left(\frac{1}{2}\Phi(c_{1-\alpha}) - \frac{1}{4}e^{-\frac{c_{1-\alpha}^2}{2}}\right)^2.$$

again using arguments as in the Appendix of Proschan and Hunsberger (1995).

References

Graf, AC., Bauer P. (2011). Maximum inflation of the type 1 error rate when sample size and allocation rate are adapted in a pre-planned interim look. *Statistics in Medicine* **30**, 1637–1647.

Proschan, MA. and Hunsberger, SA. (1995). Designed extension of Studies based on conditional power. *Biometrics* 1995; **51**, 1315–1324.
